# Twenty-Three Population-Based Trachoma Prevalence Surveys in the Central and Northern Regions of Benin, 2018–2022

**DOI:** 10.1080/09286586.2023.2265796

**Published:** 2023-11-30

**Authors:** Amadou Alfa Bio Issifou, Aboudou Dare, Gracia Adjinacou Badou, Emma M. Harding-Esch, Anthony W. Solomon, Ana Bakhtiari, Sarah Boyd, Cristina Jimenez, Anna Harte, Clara R. Burgert-Brucker, Franck Roland Sintondji, Nissou Inès Dossa, N’Koué Tatchienta Nekoua M’Po, Wilfrid Batcho

**Affiliations:** a Department of Ophthalmology, University of Parakou, Parakou, Benin; b Programme National de Lutte contre les Maladies Transmissibles, Ministère De La Santé, Cotonou, Benin; c Clinical Research Department, London School of Hygiene & Tropical Medicine, London, UK; d Global Neglected Tropical Diseases Programme, World Health Organization, Geneva, Switzerland; e International Trachoma Initiative, Task Force for Global Health, Atlanta, Georgia, USA; f Sightsavers International, Haywards Heath, UK; g Global Health Division, RTI International, Atlanta, Georgia, USA; h FHI360, Washington DC, USA

**Keywords:** Benin, prevalence surveys, trachoma, trichiasis, tropical data

## Abstract

**Purpose:**

Trachoma is an infectious eye disease caused by *Chlamydia trachomatis*. Infection causes conjunctival inflammation, which can be manifested by the sign known as trachomatous inflammation—follicular (TF). Repeated inflammation leads to eyelid scarring, which in susceptible individuals can cause in-turning of the eyelashes, referred to as trachomatous trichiasis (TT). This article describes 23 population-based surveys conducted in northern and central Benin to determine TF and/or TT prevalence for trachoma elimination purposes.

**Methods:**

A total of 18 surveys estimated the prevalence of both TF and TT: two baseline surveys, eight impact surveys after implementation of interventions against trachoma, and eight surveillance surveys. Five other evaluation units (EUs) were surveyed for TT only. To estimate the TF prevalence, a target sample size of 1701 (baseline) and 1164 1–9-year-olds (impact and surveillance) was required, whereas 2818 ≥ 15-year-olds were required to estimate the less prevalent TT. In each EU, individuals were selected by two-stage cluster sampling and examined by certified graders for TF and/or TT.

**Results:**

A total of 68,613 people were examined. TF prevalence estimates were under the 5% elimination threshold in all surveys. TT prevalence estimates were above the 0.2% elimination threshold in all five TT-only surveys and in four impact surveys, ranging from 0.2–0.57%

**Conclusion:**

TF prevalence in Benin is low, but TT was above 0.2% in nine districts. Increased case-finding and continuing efforts to improve surgery accessibility will be needed to reduce the burden of TT in Benin.

## Introduction

Trachoma is an infectious eye disease caused by *Chlamydia trachomatis*. It is characterized by inflammation of the conjunctiva.^1^ Endemic in many low- and middle-income countries,^2,3^ it may result in permanent vision loss. In the World Health Organization (WHO) simplified trachoma grading system, there are two signs of active (inflammatory) trachoma: trachomatous inflammation—follicular (TF) and trachomatous inflammation—intense (TI). Active trachoma can be treated with antibiotics,^4^ but repeated episodes of active trachoma lead to the development of eyelid scarring in susceptible individuals. This can cause in-turning of the eyelashes; which may result in corneal abrasion and the development of irreversible corneal opacity.^1^ The inturning of eyelashes due to trachoma is referred to as trachomatous trichiasis (TT) and is most commonly observed in adults over the age of 40 years, particularly women living in rural communities.^5,6^

International efforts to control trachoma have been ongoing for decades. In 1993, WHO began recommending adoption of the SAFE strategy for this purpose, incorporating four interventions: surgery, antibiotics, facial cleanliness, and environmental improvement.^7^ To support trachoma-endemic countries, in 1996, WHO launched the WHO Alliance for the Global Elimination of Trachoma by 2020 (GET2020),^8^ with the “aim of fostering planning, advocacy, research, and programme coordination towards the goal of eliminating trachoma as a public health problem by the year 2020”.^9^ Elimination is defined as a TF prevalence <5% in 1–9-year-olds, a prevalence of TT unknown to the health system <0.2% in ≥15-year-olds, and the presence of a system to identify and manage incident TT cases, in each formerly endemic evaluation unit (EU) of the country.^10^

The standardized methodology for estimating the prevalence of trachoma defines EUs as the normal administrative unit for healthcare management and a population size of 100,000–250,000 people,^11^ with teams of certified trachoma graders and data recorders then deployed to each EU to determine the prevalence of TT and TF. Surveys that are principally powered to estimate the prevalence of TF in 1–9-year-olds^12–14^ are done at baseline and at intervals after the initiation of the A, F, and E components of the SAFE strategy, with post-intervention surveys referred to as “impact” and “surveillance” surveys. (Although principally powered for TF prevalence, such surveys also involve estimation of the prevalence of TT in ≥15-year-olds.) In this article, baseline, impact, and surveillance surveys will be collectively bracketed as “standard surveys”. A TT-specific survey approach has also been developed for circumstances in which estimating TF prevalence is not needed, to ensure reliable estimates of the prevalence of this sign where that is the main index of interest.^15^ The criteria for conducting a TT-only survey relate to prevalence estimates from previous surveys, with EUs previously known to have TF prevalence <5% but TT prevalence ≥0.2% being particular candidates.

Benin is a country in West Africa bordered by Nigeria, Togo, Burkina Faso, and Niger. All of these countries are currently or previously trachoma-endemic.^16–22^ Between 2014 and 2015, the National Program for the Control of Communicable Diseases (PNLMT) mapped (as 11 EUs) all 26 districts suspected of being endemic for trachoma.^23^ There has been no strategic implementation of the F and E aspects of SAFE in Benin. The WHO/UNICEF joint monitoring programme (JMP) estimated that in 2018, the national proportion of households with access to potable drinking water was ~66%, safely managed sanitation was ~16%, and basic hygiene services was ~11%.^24^

At the time of the baseline surveys, eight districts (four EUs) had a TF prevalence in 1- to –9-year-olds ≥5%. In accordance with WHO recommendations, the PNLMT implemented three rounds of antibiotic mass drug administration (MDA) in the four districts (two EUs) that had a TF prevalence ≥10% but <30%, and one round of MDA in the four districts (two EUs) that had a TF prevalence ≥5% but <10%. EUs that undergo MDA should subsequently undergo an impact survey to determine whether MDA can be safely stopped.^25^ If the TF prevalence at impact survey is below the 5% threshold, surveillance surveys should be conducted at least 2 years later to ensure that the low prevalence has been maintained without further MDA.^25^ When both TF and TT elimination thresholds have been met in all endemic EUs, countries can apply for validation of trachoma elimination.^10^ In addition to demonstrating trachoma elimination in EUs known to have been previously endemic, countries must provide evidence that areas that were not suspected to be endemic do not have a public health problem from trachoma. In 2022, therefore, Benin’s Ministry of Health conducted additional baseline surveys in Ouèssè and Bantè, as these districts separate the trachoma-endemic northern region from the southern region that is understood to be trachoma-free.

The baseline surveys in 2014 and 2015 showed that 19 districts (nine EUs) had a prevalence of trichiasis above 0.2%, ranging from 0.3% to 1.9%, with an estimated total case count of >11,000 people.^23^ Of these 19 districts, 11 (comprising five EUs) fulfilled the criteria for requiring a subsequent TT-only survey^15^ because they formed EUs that had TF prevalence <5% but TT prevalence ≥0.2%. Due to the expense associated with conducting surveys^26^ and limited funding available, only five districts, each treated as a separate EU, were chosen for TT-only surveys in the present tranche of work: Kérou, Kouandé, Péhunco, Bembèrèkè, and Sinendé, all located in Benin’s northern region. In the remaining six districts, the PNLMT undertook house-to-house TT case-finding in order to get full geographical coverage of TT screening^27^; the data from those exercises are not presented here.

This article describes the methodology and results of 18 standard and 5 TT-only surveys conducted in Benin from 2018 to 2022.

## Materials and methods

### Study ethics and consent

The objectives were explained to each village chief, the head of each household, and each individual participant in the local language before obtaining written consent, with the parent/guardian giving consent on behalf of children aged 1–14 years. Individuals who had TF were given 1% tetracycline ophthalmic ointment, and those with TT unknown to the health system were referred to PNLMT TT surgeons for management free of charge. Ethical approval was provided by the National Ethics Committee for Health Research (CNERS), Benin, and the London School of Hygiene & Tropical Medicine, UK (reference: 16105).

### Study design and participant selection

The methodology is used to select the number of villages and households used two-phase cluster sampling.^13–15^ Local elected representatives and/or community guides were consulted to determine how to segment clusters into groups of households.

#### Sample size and cluster calculations

To estimate the prevalence of TF and TT at EU level, the sample size was calculated using the expected prevalence for the survey type (baseline surveys: TF = 10%, impact and surveillance surveys: TF = 4%, TT-only surveys: TT = 0.2%), the desired absolute width of the 95% confidence interval (baseline surveys: TF = 3%, impact and surveillance surveys: TF = 2%, TT-only surveys: TT = 0.2%), a design effect (baseline surveys = 3.69, impact and surveillance surveys = 2.63, TT-only surveys = 1.47),^28^ and a non-response rate inflation multiplier of 1.2 (to account for absence, refusal, and inability to participate).^13^ Using the formula for a single population proportion for precision,^29^ a minimum sample size of 1164 children aged 1–9 years was required to be enumerated in impact and surveillance survey EUs, 1701 children aged 1–9 years were required in baseline EUs and 3382 adults aged ≥15 years were required for TT-only EUs. Because the population of Toucountouna was less than 100,000, a finite population correction factor was applied for the sample size calculation there, and the number of children to be enumerated was 1145.

Survey teams can reliably visit 25 households per day for the standard surveys and 30 households for the TT-only surveys. To calculate the number of clusters required to meet the minimum sample size for the standard surveys, the mean number of the target population (standard surveys: children aged 1–9 years, TT-only surveys: adults aged ≥15 years) per household was multiplied by the number of households a team can visit (standard surveys = 25, TT-only surveys = 30), and then the required sample size was divided by this value (Table 1). For TT-only this resulted in 36 clusters of 30 households needed per EU; in-line with WHO recommendations, however, 30 clusters were surveyed, as little additional precision around a TT prevalence estimate is expected from surveying >30 clusters.^15^

### Definitions

the WHO’s simplified trachoma grading system^30,31^ defines TF as the presence of five or more follicles, each at least 0.5 mm in diameter, in the central part of the upper tarsal conjunctiva.

The definition of TT in the simplified trachoma grading system changed during the implementation of the impact and surveillance surveys. Version 1 surveys, completed in 2018–2019, used the original definition of TT, which states that TT is the presence of at least one eyelash from the upper or lower eyelid touching the eyeball, or evidence of recent epilation of in-turned eyelashes.^31^ The 4th Global Scientific Meeting on Trachoma (GSM4) held in 2018 redefined TT as the presence of one or more eyelashes from the upper eyelid touching the eyeball, or evidence of recent epilation of in-turned eyelashes from the upper eyelid.^32^ This change was due to the fact that diseases other than trachoma cause in-turning of the eyelids. Subsequent version 2 surveys (including all the TT-only surveys reported here) used this definition. As a result of this definition modification, cases of TT classified before the changes were implemented are described in this article as cases of trichiasis, rather than TT.

### Grader/Recorder training and clinical examinations

Prior to the start of the surveys, graders were trained to identify TF and/or TT (the latter according to the definition in use at the time.^30,31^ Standard survey training involved 2–3 days of theory and field training, with graders assessed through two Inter-Grader Agreement (IGA) tests; in the first, graders were required to obtain a kappa of ≥0.7 when grading TF from a set of 50 photographs, in the second they were required to obtain the same kappa score when grading TF from 50 children.^33^ Successful graders continued to team training including half a day of field practice. TT-only survey training involved 2 days of theory and one of field practice, using the methods codified by Tropical Data (www.tropicaldata.org).^34^ At the end of the training, graders were assessed using an Objective Structured Clinical Evaluation: a structured, standardized system for evaluating clinical skills. In both survey-type trainings, recorders were trained on how to enter and upload data using Android smartphones and the Tropical Data app. This included recorder reliability tests to ensure that recorders were recording all survey information accurately, with a required 90% pass rate. The same 16 graders were used throughout the survey period, and all were trained and certified prior to the start of the COVID-19 pandemic. Graders underwent retraining in 2020 to incorporate GSM4 updates, via live online Tropical Data training courses. Refresher training sessions were also conducted prior to every survey; these were also held online during the pandemic as in-person training was not possible due to travel limitations. COVID-19-related training updates included the addition of face-shields during examinations, maintaining the recommended safe distance wherever possible and enhanced hand-washing.^35^

Graders used 2.5× magnifying loupes and torches to examine each eye for TF, TI, and/or trichiasis. This involved inspection with a torch to see if any eyelashes came into contact with the eyeball or if there was evidence of recent removal of in-turned eyelashes, and everting the eyelid to check the upper tarsal conjunctiva for TF. From 2019 onwards, self-adhesive guides for evaluating the size of the follicles were used to improve TF diagnosis accuracy.^36^ If trichiasis was present in the upper or lower eyelid, the grader would ask the individual about any prior surgery or epilation that they had been offered by a health professional, to determine whether the health system was aware of the presence of trichiasis in that eye. From 2019 onwards, if participants indicated that they had undergone trichiasis surgery, graders looked for evidence of surgical scars to confirm surgical intervention.

### Water, sanitation, and hygiene access

In standard surveys, the head of each household was asked questions on water, sanitation, and hygiene (WASH) access. These included questions on latrine type, the source of washing and drinking water, distance to latrines and water, and the availability of soap. TT-only surveys did not include the WASH data component.

## Data analysis

Data were uploaded to the Tropical Data server. Once all data from all clusters sampled in the EU were collected and cleaned, the prevalence of TF and/or TT was estimated using previously described methods, adjusting for age and gender based on population census estimates.^13,37^ Confidence intervals were constructed using bootstrapping with a resampling-replacement method spanning 10,000 replicates. We performed association analyses using mixed effects regression models to determine odds ratios (OR) for WASH variables, age and gender on the likelihood of finding TF, and OR for age and gender on the likelihood of finding TT, using EU, cluster, and household as random effects. For the purposes of the TF association analyses, ages were grouped as 1–6, 7–15, and 16+ years. While TF prevalence estimates are based on children aged 1–9 years, individuals aged ≥10 were included in the association analyses as older children and adults, particularly women, can still have active disease and contribute to the spread of ocular *C. trachomatis* infection.^38–40^ Large age brackets were used because TF numbers were low, and all WASH variables were converted to binary variables (e.g., improved/unimproved) in order to ensure enough TF cases were present in each group. The TT association analyses used the age groups 15–45 years, 46–75 years, and ≥76 years. The 5-year age bins for TT recommended by Macleod et al.^41^ were not suitable due to the low number of TT cases present in younger age groups.

## Results

### Populations

#### Standard surveys

Between 2018 and 2022, we conducted two baseline surveys, eight impact surveys, and eight surveillance surveys. The eight surveillance EUs were all conducted in EUs for which impact surveys are also described in this series. A total of 56,144 people were enumerated 27,405 (48.8%) of which were children aged 1–9 years. About 273,50 children (99.8%) were examined, with 20–30 clusters included and 1214–1931 children examined per EU (Table 2).

#### TT-only surveys

We conducted fieldwork in March 2020. A total of 13,711 people aged ≥15 years were enumerated, with 13,435 (98%) examined (Table 3). Women represented 59% of those examined. In each EU, ~900 households and 30 clusters were included, with the number of people examined per EU ranging from 2,307 to 3,060.

## Clinical findings

### Standard surveys

**Figure 1:**
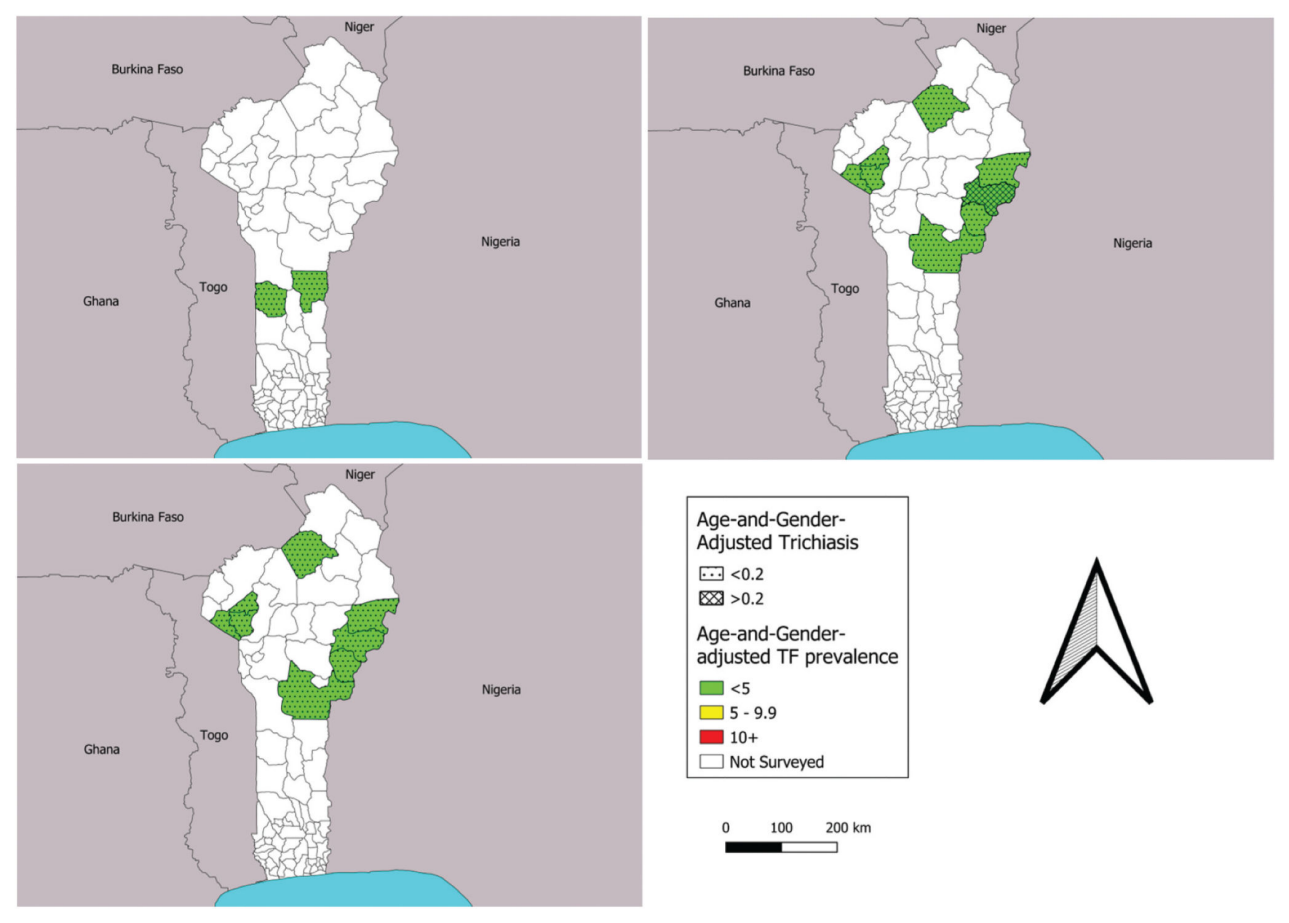
Prevalence of trachomatous inflammation—follicular (TF) and trichiasis at (a) baseline, (b) impact and (c) surveillance surveys conducted in Benin, 2018–2022. The boundaries and names shown and the designations used on this map do not imply the expression of any opinion whatsoever on the part of the authors, or the institutions with which they are affiliated, concerning the legal status of any country, territory, city or area or of its authorities, or concerning the delimitation of its frontiers or boundaries.

**Figure 2:**
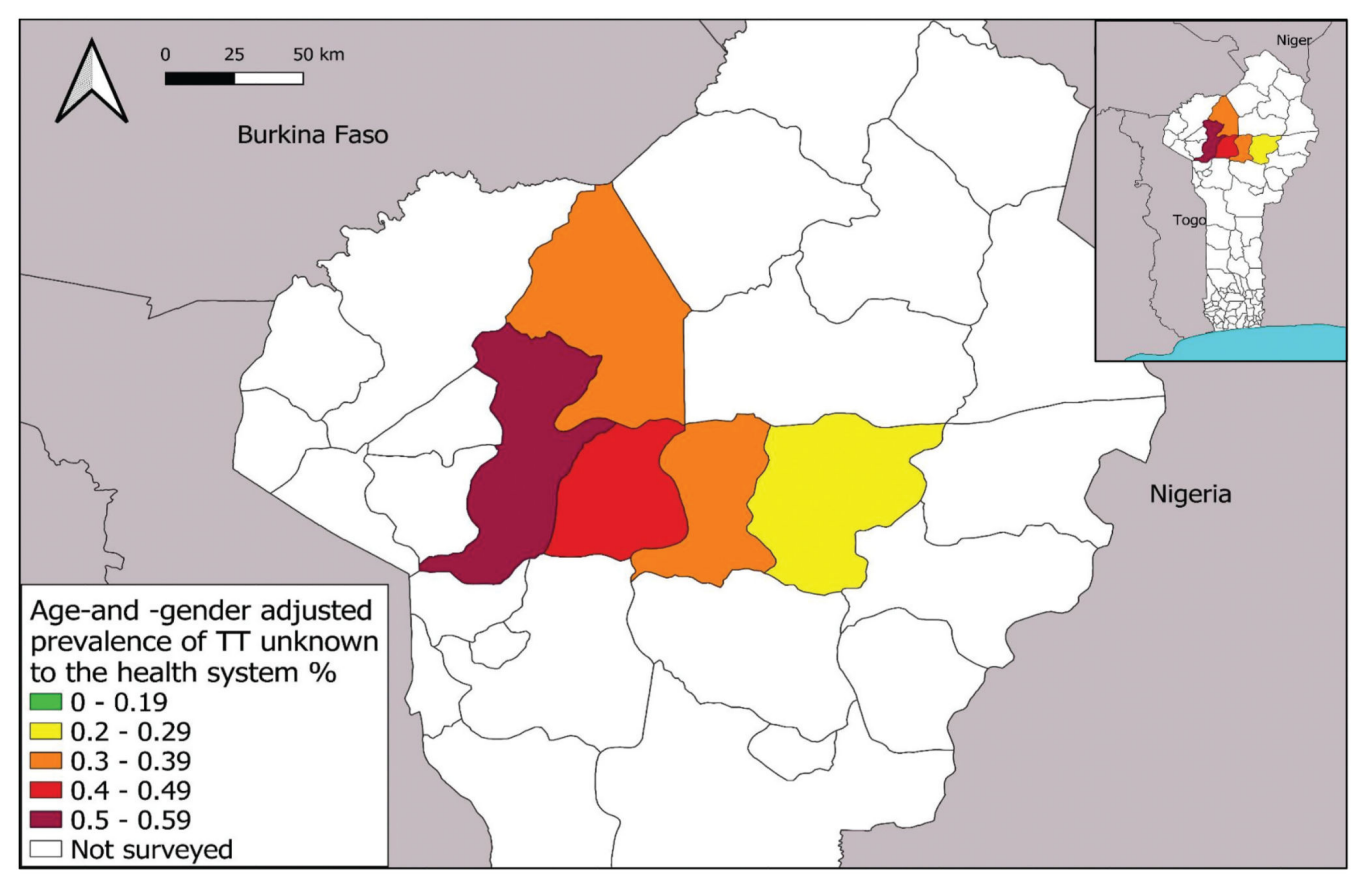
Age- and gender-adjusted prevalence of trachomatous trichiasis (TT) unknown to the health system in five surveyed evaluation units, Benin, March 2020. The boundaries and names shown and the designations used on this map do not imply the expression of any opinion whatsoever on the part of the authors, or the institutions with which they are affiliated, concerning the legal status of any country, territory, city or area or of its authorities, or concerning the delimitation of its frontiers or boundaries.

TF prevalence estimates were below the 5% elimination threshold in all 18 standard surveys (Table 2, Figure 1). The highest prevalence estimate of 2.5% was in Pèrèrè in 2018, and the lowest was 0%, recorded on seven occasions. All surveillance surveys had a lower TF prevalence estimate than the impact survey estimate generated previously in the same EU. Graders found a total of 159 cases of TF in 1–9-year-olds in the 18 surveys combined. Age- and gender-adjusted trichiasis prevalence estimates unknown to the health system from version 1 impact surveys ranged from 0 to 0.39%, with four EUs above the 0.2% threshold (Table 2). Similarly adjusted TT estimates from the version 2 baseline and surveillance surveys ranged from 0 to 0.03%, with all below the elimination threshold (Table 2, Figure 2).

### TT-only surveys

The age- and gender-adjusted estimates of the prevalence of TT unknown to the health system ranged from 0.21% to 0.57% for the five EUs, with the lowest rate in Bembèrèkè, and the highest in Kouandé, in the north-western region of Benin (Table 3, Figure 2). In total, 114 people had TT. There were two cases of lower eyelid-only trichiasis.

## WASH data

The majority (77%) of households had access to an improved drinking water source, and 62% of households could access water within 30 min (Table 2). A mean of 12% of people had access to an improved latrine, with EU-level estimates ranging from 3% in Natitingou (2021, surveillance survey) to 35% in Bantè (2022, baseline survey).

## Association analyses for TF

Household alone was found to be the most appropriate random effect. The TF association analysis did not identify evidence of associations of TF with any WASH variable (Table 4). There was, however, strong evidence of an association between age and gender with TF, with people aged over 16 years having lower odds of having TF than children aged 1–6 years (*p* < .001, AOR: 0.14, CI: 0.08–0.18). Females were also more likely than males to have TF (*p* < .01, AOR: 1.8, CI: 1.19–2.81).

## TT age and gender association analysis

TT data from standard prevalence surveys were added to the TT-only dataset, excluding data from version 1 surveys due to differences in TT definitions as previously described. EU, cluster, and household were included as random effects both separately and in combination, but EU alone had the lowest Akaike’s information criteria (AIC) value, and so the other random effects were removed from the final models. Age and gender were both highly associated with the presence of any TT (including cases known and unknown to the health system). Relative to people aged 15–45 years, people aged 46–75 years were 49 times more likely to have TT (*p* < .001, AOR: 48.5, CI: 23–104) and people aged ≥76 years were 155 times more likely to have TT (*p* < .001, AOR: 155.2, CI: 69–351). Women were 3.3 times more likely than men to have TT (*p* < .001, AOR: 3.3, CI: 2.2–5.2).

## Discussion

The results of both the standard surveys and TT-only surveys demonstrate that while TF prevalence is below the elimination threshold across all endemic EUs, TT remains a public health issue in the central northern region of Benin.

TF prevalence decreased (or remained at zero) between impact survey and surveillance survey for each EU surveyed twice in this series, demonstrating a sustained low level of TF (Figure 1). Benin has invested a significant amount of time and resources in treating trachoma endemic areas since the baseline surveys in 2014–2015 revealed high TF prevalences,^23^ and it is clear that these efforts have been successful. The TT estimates from the TF prevalence surveys also suggest that in trachoma endemic areas, TT service provision is working, as the EU with the highest trichiasis estimate, Nikki, saw an unmanaged trichiasis prevalence decrease from a 0.39% at impact survey to 0.0% at surveillance survey (Table 2, Figure 2). Ongoing intervention plans for TT in Benin include door-to-door TT case-finding, mobile clinics, for which ophthalmologists and/or senior ophthalmology technicians are trained to operate on TT cases in the community, as well as the use of a new TT-tracker app to track TT cases and surgical outcomes for individuals.^42,43^ Overall, trichiasis prevalence has fallen across the board since the 2014–2015 baseline surveys; nine EUs were above 0.2% at baseline, whereas only four remain above 0.2% now (Table 2). While these data are taken from surveys powered to detect TF and not TT, they do suggest that TT treatment is being accessed. It is, however, also possible that the reduction is partly due to the change in the definition of TT between the two time points.

All five TT-only surveys returned TT prevalence above the elimination threshold of 0.2% in ≥15-year-olds (Figure 2).^10^ Baseline survey data generated in 2014–2015 used just two EUs (Kouandé–Kérou – Péhunco [number of ≥15-year-olds examined = 1425 from 23 clusters, each of 30 households] and Sinendé–Bembèrèkè [*n* = 1754; 23 clusters of 30 households]); the estimated TT prevalence in each of them was 0.3%, with each estimate having 95% confidence intervals of 0.1–0.7%. The later surveys reported here suggest that the TT prevalence unknown to the health system may be up to two-fold higher (0.57%, CI: 0.32–0.89%) in Kouandé than the 0.3% baseline estimate for Kouandé–Kérou – Péhunco combined.

A common issue associated with TT surgery in Benin is accessibility, as many rural locations are a significant distance from central hospitals. As TT is associated with older age and visual impairment, it is likely that patients will need to transport to and from the place where surgery can be provided. Studies have shown that with increased case-finding efforts and provision of transport, the proportions of people accepting and receiving surgical intervention increase significantly.^44–46^ Improved case-finding, and the provision of either transport to and from treatment centers or more local surgical sites, will help reduce the prevalence of TT unknown to the health system in each Benin EU towards the elimination threshold.

Our analyses demonstrated that age and gender were significantly associated with the prevalence of both TT and TF, with older women much more likely than younger men to have TT, and younger girls more likely than boys to have TF. Previous studies elsewhere have shown that women carry a disproportionate risk of having TT,^5,38,47,48^ and a number have shown TF to be more prevalent in young girls.^48–50^ This may be due to women and young girls spending more time than men or boys in close proximity to (other) children, the principal reservoir of ocular *C. trachomatis* infection.^6,38^

The strengths of our surveys lie in the standardized methodologies and quality control and quality assurance measures taken.^51^ We acknowledge that the TT-only estimated sample size of 2818 people per EU was not reached in three of the five surveyed TT-only EUs. However, the WHO recommends that including 30 clusters with at least 30 households per cluster provides sufficient precision around TT prevalence estimates,^15^ and a balance must be struck between precision and the time and financial cost of surveys. We also acknowledge that the preponderance of women in the group examined suggests under-representation of men despite the apparently high response rate, however prevalence estimates are adjusted against the underlying population to account for imbalances in recruitment between genders.

Benin has been engaged in treating active trachoma using population-wide antibiotic MDA since 2016, and as demonstrated in the results of the impact and surveillance surveys here, all surveyed districts have a TF prevalence in 1–9-year-olds below the elimination threshold of 5%. This reduction in active trachoma will likely reduce the future incidence of TT, but prevalent cases of TT require individual management as part of a public health response.

## Figures and Tables

**Table 1:** Values used to calculate the sample size and clusters required for each evaluation unit (EU) and survey type.

EU	Survey type	Mean number of target population per household	Design effect	Expected prevalence %	Minimum sample size	Number of clusters required
Ouèssè	Baseline	1.62	3.69	10	1701	30
Bantè	Baseline	1.62	3.69	10	1701	30
Banikoara	Impact	2.83	2.63	4	1164	20
Natitingou	Impact	2.43	2.63	4	1164	20
Boukoumbé	Impact	2.43	2.63	4	1164	20
Toucountouna	Impact	2.43	2.63	4	1139	20
Nikki	Impact	2.1	2.63	4	1164	24
Kalale	Impact	2.1	2.63	4	1164	24
Pèrèrè	Impact	2.1	2.63	4	1164	24
Tchaourou	Impact	2.1	2.63	4	1164	24
Banikoara	Surveillance	2.83	2.63	4	1164	20
Natitingou	Surveillance	2.43	2.63	4	1164	20
Boukoumbé	Surveillance	2.43	2.63	4	1164	20
Toucountouna	Surveillance	2.43	2.63	4	1145	20
Nikki	Surveillance	2.1	2.63	4	1164	23
Kalalé	Surveillance	2.1	2.63	4	1164	23
Pèrèrè	Surveillance	2.1	2.63	4	1164	23
Tchaourou	Surveillance	2.1	2.63	4	1164	23
Kérou	TT-only	3.1	1.47	0.2	3382	30
Kouandé	TT-only	3.1	1.47	0.2	3382	30
Péhunco	TT-only	3.1	1.47	0.2	3382	30
Bembèrèkè	TT-only	3.1	1.47	0.2	3382	30
Sinendé	TT-only	3.1	1.47	0.2	3382	30

**Table 2:** Standard survey demographics, trachomatous inflammation—follicular (TF) prevalence, trachomatis trichiasis (TT) prevalence and access to water, sanitation, and hygiene in 10 evaluation units (EUs), Benin, 2018–2021. CI: confidence interval.

County	District	EU ID	Date	Survey type*	No. Clusters surveyed	No. Households surveyed	No. 1–9-year-olds Examined (% enumerated)	Age-adjusted TF prevalence (95% Cl)	Age- and gender-adjusted prevalence of TT unknown to the health system (95% Cl)	Age- and gender-adjusted prevalence of trichiasis unknown to the health system (95% Cl)	Percentage of surveyed households with improved drinking water source	Percentage of surveyed households with drinking water source within 30 minutes	Percentage of surveyed households with an improved latrine
Alibori	Banikoara	80977	2019	impact v1	24	614	1518 (99.7)	0.16 (0–0.41)		0.04(0–0.12)	10.9	89.7	6.2
	Banikoara	60534	2021	surveillance	20	603	1229 (100)	0	0		44.3	60.0	13.1
				v2									
Collines	Bantè	20041	2022	baseline v2	30	750	1566 (99.9)	0	0		96.0	73.1	34.9
Atacora	Boukoumbé	80979	2019	impact v1	24	607	1418 (99.7)	0.50 (0.07–0.84)		0.02 (0–0.06)	79.4	46.0	4.4
	Boukoumbé	60535	2021	surveillance	20	599	1214(100)	0	0		72.5	39.9	5.0
				v2									
Borgou	Kalalé	80723	2018	impact v1	24	622	1484(99.2)	1.14(0.48–2.03)		0.31 (0.06–0.68)	72.7	71.5	14.3
	Kalalé	60538	2021	surveillance	23	689	1520 (99.9)	0.74 (0.32–1.29)	0.03 (0–0.10)		93.0	78.7	12.3
				v2									
Atacora	Natitingou	80978	2019	impact v1	24	619	1405 (99.7)	0		0.08 (0–0.22)	79.5	80.0	8.6
	Natitingou	60536	2021	surveillance	20	604	1242 (99.8)	0	0		75.8	63.6	3.0
				v2									
Borgou	Nikki	80724	2018	impact v1	24	616	1931 (99.9)	1.94(1.35–2.66)		0.39 (0.12–0.78)	94.0	34.4	18.7
	Nikki	60539	2021	surveillance	23	690	1669 (99.9)	0.07 (0–0.17)	0		93.0	64.6	12.8
				v2									
Collines	Ouèssè	20042	2022	baseline v2	30	753	1869 (99.8)	0.06 (0–0.18)	0		96.1	91.2	23.4
Borgou	Pèrèrè	80725	2018	impact v1	24	611	1877 (99.9)	2.49 (1.48–3.30)		0.21 (0.02–0.49)	97.7	15.2	4.9
	Pèrèrè	60540	2021	surveillance	23	693	1826 (99.7)	0.38 (0.04–0.80)	0.02 (0–0.07)		83.1	50.8	17.2
				v2									
	Tchaourou	80726	2018	impact v1	24	632	1463 (99.7)	0.67 (0.28–1.18)		0.20 (0–0.47)	85.1	69.8	11.2
	Tchaourou	60541	2021	surveillance	23	692	1424(100)	0.54 (0.24–0.95)	0.02 (0–0.07)		96.4	58.2	19.9
				v2									
Atacora	Toukountouna	80980	2019	impact v1	24	602	1384 (99.4)	0		0.04(0–0.12)	76.2	74.6	4.8
	Toukountouna	60537	2021	surveillance	20	600	1311 (100)	0	0		41.8	59.7	9.2
				v2									

* v1 refers to version 1 of the Tropical Data trachoma prevalence survey methodology used in surveys conducted prior to 2019. v2 surveys used the post-2019 version 2 methodology.

**Table 3:** Survey demographics and trachomatous trichiasis (TT) prevalence in five evaluation units (EUs), Benin, March 2020. CI: confidence interval; TF: trachomatous inflammation—follicular.

County	District	EU ID	2014-2015 survey baseline prevalence estimates*		No. Clusters selected	No. Households surveyed	Number of people aged ≥15 years examined (enumerated response rate, %)	Number of people aged ≥15 years examined who were female (%)	Number of people aged ≥15 years with TT	Number of people aged ≥15 years with TT + TS	Age-and gender adjusted prevalence of TT in those aged ≥15 years (95% Cl)	Age- and gender-adjusted prevalence of TT unknown to the health system in those aged ≥15 years (95% Cl)
			TF % in 1–9-year-olds (95% Cl)	TT %in ≥15-year-olds 95% Cl								
Atacora	Kérou	50164	4.5 (3.3–5.9)	0.3 (0.1–0.7)	30	903	2307 (99)	1207 (52)	18	15	0.30 (.13–53)	0.30 (0.13–0.52)
	Kouandé	50165	4.5 (3.3–5.9)	0.3 (.1–7)	30	903	2775 (94)	1632 (59)	27	23	0.63 (.36–.99)	0.57 (.32–.89)
	Péhunco	50166	4.5 (3.3–5.9)	0.3 (.1–7)	30	901	2818 (99)	1783 (63)	25	15	0.44 (.24–.70)	0.42 (.23–.67)
Borgou	Bembèrèkè	50167	2.7 (1.4–4.5)	0.3 (0.1–0.7)	30	902	2475 (98)	1528 (62)	18	17	0.31 (.14– 56)	0.21 (0.07–0.43)
	Sinendé	50168	2.7 (1.4–4.5)	0.3 (.1–7)	30	899	3060 (99)	1814(59)	26	16	0.42 (.21–.69)	0.37 (.16–.63)

**Table 4:** Association analyses for trachomatous inflammation—follicular. OR: odds ratio. CI: confidence interval.

Variable	Value	Univariable OR (95% CI)	Univariable P value	Multivariable OR (95% CI)	Multivariable P value
Age (years)	1–6	Reference	-	-	-
	7–15	0.66 (0.42–1.02)	0.06	0.52 (0.24–0.96)	0.06
	16+	0.09 (0.05–0.17)	<0.001	0.14 (0.08–0.18)	<0.001
Gender	Male	Reference	-	-	-
	Female	1.52 (1.02–2.19)	0.04	1.8 (1.19–2.81)	0.005
Latrine type	Unimproved	1.28 (0.04–46.36)	0.89	-	-
	Improved	Reference	-	-	-
Drinking water source	Unimproved	0.51 (0.01–16.54)	0.71	-	-
	Improved	Reference		-	-
Distance to drinking water	≤30 minutes	0.22 (0.01–6.13)	0.37	-	-
	>30 minutes	Reference		-	-
Handwash station	Present in yard	3.82 (0.03–392.61)	0.57	-	-
	Not present in yard	Reference	-	-	-
Number of children aged 1–9 years per household	0–1	Reference	-	-	-
	2–5	2.90 (2.68–315.07)	0.66	-	-
	6+	5.81 (2.63–128.11)	0.52	-	-
